# *Klebsiella pneumoniae*–Induced Liver Abscesses, Germany

**DOI:** 10.3201/eid2011.140149

**Published:** 2014-11

**Authors:** Sueleyman Bilal, Magdalena Sarah Volz, Tomas Fiedler, Rainer Podschun, Thomas Schneider

**Affiliations:** Charité University Medicine Berlin, Berlin, Germany (S. Bilal, M.S. Volz, T. Schneider);; Rostock University Medical Center, Rostock, Germany (T. Fiedler);; University Hospital Schleswig-Holstein, Kiel, Germany (R. Podschun)

**Keywords:** Klebsiella pneumoniae, liver abscess, community-acquired, emerging, bacteria

**To The Editor:** Monomicrobial liver abscesses caused by *Klebsiella pneumoniae* are an emerging problem in Asia. Among 77 capsular types of *Klebsiella* spp., K1 and K2 are the most virulent (*1*–*3*). In Asia, isolated strains are mainly serotype K1, followed by K2. In Taiwan, about 63.4% of liver abscesses caused by *K. pneumoniae* are associated with K1 strains and 14.2% by K2 (*4*); no comparable epidemiologic data for Western countries are available (*5*,*6*). Cases of *K. pneumoniae* liver abscesses have been also reported from North America and Europe (*6*–*9*); patients outside of Asia predominantly have a history of traveling to high-prevalence countries and/or Asian ethnicity (*10*). 

We describe 2 cases of *K. pneumoniae* liver abscesses in patients in Germany. Both cases were associated with life-threatening metastatic spread of *K. pneumoniae* in previously healthy white patients with no travel history, no Asian ethnicity, and no contact with persons in high-risk groups, including patients with *K. pneumoniae* infection.

Patient 1 was a 48-year-old white male medical doctor with no history of serious medical conditions, who suddenly experienced fever up to 40°C, abdominal pain, mild diarrhea, and fatigue. Blood analysis revealed signs of inflammation: C-reactive protein level of 26 mg/dL. Erythrocyte and leukocyte counts were within reference ranges, but thrombocytes were decreased (43,000 cells/μL) and liver enzymes were increased (glutamate pyruvate transaminase 112 U/L, aspartate aminotransferase 75 U/L, gamma glutamyl transferase 332 U/L). An initial chest radiograph and abdominal ultrasonograms were unremarkable. *K. pneumoniae* was isolated from blood and urine and was later characterized as K1 capsular type (no further typing techniques could be performed because no sample was stored). Ceftriaxone was administered, in accordance with an antibiogram, but the patient’s condition worsened and he required mechanical ventilation and treatment with a catecholamine in an intensive care unit. A computed tomographic (CT) scan showed 2 liver abscesses and infiltrates of both lungs with pleural effusion (Figure). Under CT guidance, the liver abscesses were punctured and drained percutaneously by using pigtail catheters. After this intervention, the patient recovered gradually. The drain was removed after 10 days, and ceftriaxone treatment was continued for a total of 21 days. The patient recovered and was transferred to a rehabilitation hospital.

**Figure:**
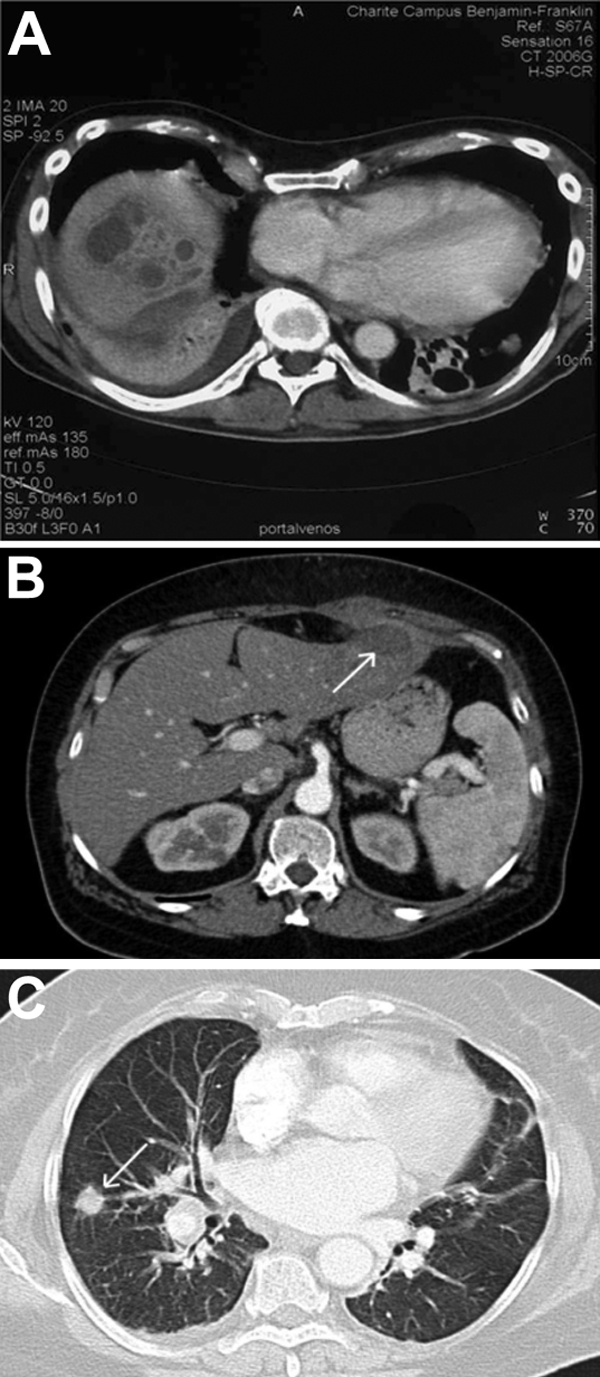
Figure. Pretreatment computed tomography images of patients with *Klebsiella pneumoniae* liver abscesses. A) Multilobular abscess in segment 8 of the liver and pleural effusion in 48-year-old white man (patient 1). B) Liver abscess (arrow) in 71-year-old white woman with type-2 diabetes (patient 2). C) Lung lesion (arrow) in patient 2.

Patient 2 was a 71-year-old white woman with type-2 diabetes, who was hospitalized for epigastric pain and fatigue. Laboratory results showed an increased level of C-reactive protein (13 mg/dL), blood count and liver enzymes within reference range, and a urinary tract infection positive for nitrite and leukocytes (500/μL). Abdominal ultrasonography revealed a 3 × 4-cm subcapsular lesion in the left lobe of the liver, highly suspect for metastatic spread of an unknown tumor. Chest radiographs, taken in search of the primary lesion, revealed a lesion in the right upper lung; on follow-up CT images, this lesion was suspect for malignancy (Figure). For diagnostic purposes, the liver lesion was punctured and a sample was obtained. Histologic analysis revealed pus, which was in accordance with an abscess. A pigtail drain was placed, and *K. pneumoniae* was cultured from the liver punctate as well as from the urine. The isolate proved to be serotype K2. Multilocus sequencing of this strain confirmed the presence of the *wzx2* and *wzy2* genes. In accordance with susceptibility test results, therapy with ceftriaxone and ciprofloxacin was initiated. Dislocation of the pigtail catheter resulted in an abscess of the abdominal wall, which required additional surgical treatment. However, the patient recovered within 2 weeks. Follow-up CT images showed resolution of the thoracic lesion and only a residual scar on the liver lobe (Technical Appendix). 

These 2 cases of community-acquired *K. pneumoniae* serotype K1 and K2 liver abscesses with metastatic spread to the lung and urinary systems in previously healthy white patients from Germany differ from previously published cases. These 2 patients were not of Asian ethnicity and had no travel history, no contact with persons in a high-risk group (*10*), and no common risk factors such as malignancy (*8*); however, 1 patient had type-2 diabetes. *K. pneumoniae* liver abscesses might be an emerging problem with global spread. Although initial radiographic findings might more commonly indicate metastasis than abscesses, differential diagnosis of liver lesions should include *K. pneumoniae–*induced abscesses. 

Technical Appendix Posttreatment computed tomography images for 71-year-old white woman with type-2 diabetes (patient 2) and PCR of the *Klebsiella pneumoniae* K2 strain.
