# Supplementary material for: Klebsiella pneumoniae–Induced Liver Abscesses, Germany
Source: Emerg Infect Dis. 2014 Nov;20(11):1939–40. doi: 10.3201/eid2011.140149 (PMC4214298; doi:10.3201/eid2011.140149)
Supplement: Technical Appendix — Posttreatment computed tomography images for 71-year-old white woman with type-2 diabetes (patient 2) and PCR of the Klebsiella pneumoniae K2 strain. [file 14-0149-Techapp-s1.pdf]

Article DOI: http://dx.doi.org/10.3201/eid2011.140149

Klebsiella pneumoniae–Induced Liver Abscesses, Germany

Technical Appendix

A

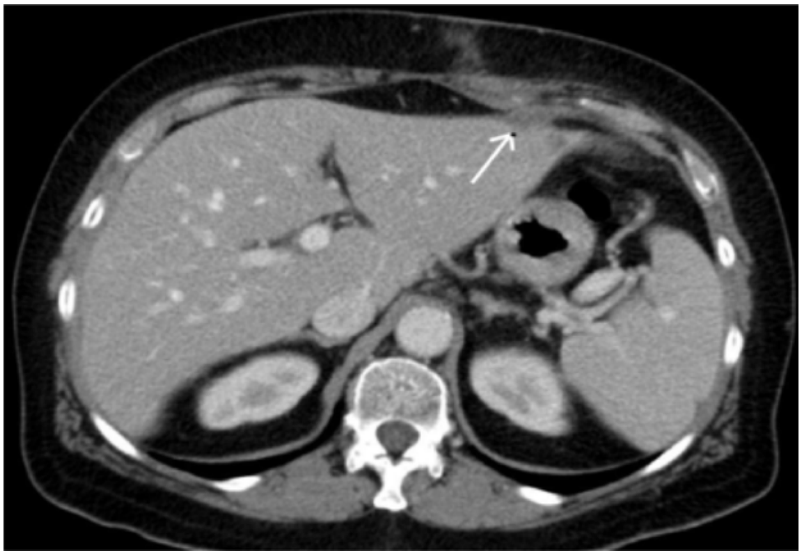

B

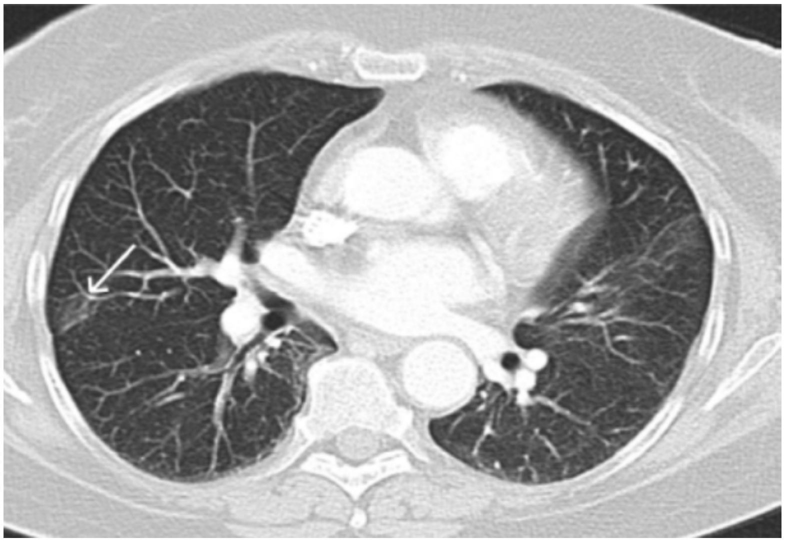

C

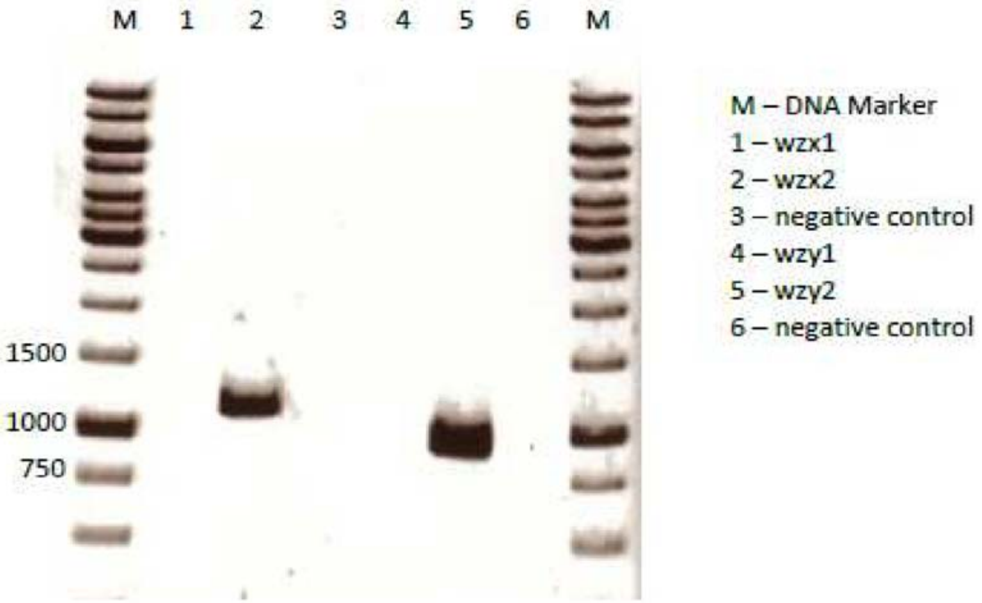

Technical Appendix legend. A and B) Posttreatment computed tomography images of liver abscess (arrow) (A) and lung lesion (B) in 71-year-old white woman with type-2 diabetes (patient 2). C) PCR of the *Klebsiella pneumoniae* K2 strain isolated from a patient with liver abscess (patient 2).
